# Psychological Health in the Retirement Transition: Rationale and First Findings in the HEalth, Ageing and Retirement Transitions in Sweden (HEARTS) Study

**DOI:** 10.3389/fpsyg.2017.01634

**Published:** 2017-09-26

**Authors:** Magnus Lindwall, Anne Ingeborg Berg, Pär Bjälkebring, Sandra Buratti, Isabelle Hansson, Linda Hassing, Georg Henning, Marie Kivi, Stefanie König, Valgeir Thorvaldsson, Boo Johansson

**Affiliations:** Department of Psychology, University of Gothenburg, Gothenburg, Sweden

**Keywords:** aging, retirement, longitudinal study, cohort study, transition

## Abstract

From an aging research and life-course perspective, the transition to retirement marks a significant life-event and provides a unique opportunity to study psychological health and coping during a period of substantial change in everyday life. The aim of the present paper is to: (a) outline the rationale of the HEalth, Ageing and Retirement Transitions in Sweden (HEARTS) study, (b) describe the study sample, and (c) to present some initial results from the two first waves regarding the association between retirement status and psychological health. The HEARTS study is designed to annually study psychological health in the years before and following retirement, and to examine change and stability patterns related to the retirement event. Among a representative Swedish population-based sample of 14,990 individuals aged 60–66 years, 5,913 completed the baseline questionnaire in 2015. The majority of the participants (69%) completed a web-based survey, and the rest (31%) completed a paper version. The baseline HEARTS sample represents the general population well in terms of gender and age, but is more highly educated. Cross-sectional findings from the first wave showed that retired individuals demonstrated better psychological health compared to those who were still working. Longitudinal results from the first and second waves showed that individuals who retired between waves showed more positive changes in psychological health compared with those still working or previously retired.

## Introduction

The retirement event signals the entry into the third age (Laslett, 1991). The period of transition into retirement offers a unique time window that allows researchers to study adaption and coping over a period characterized by substantial everyday-life changes that may affect overall psychological health. Early research on the retirement transition has largely focused on financial conditions and physical health and less on psychological aspects, but this trend has changed in recent years as more interest in psychological factors has been noted (Shultz and Wang, 2011). However, there are still substantial gaps in the current literature on how individuals experience and cope with the retirement transition (Shultz and Wang, 2011; Wang et al., 2011). As emphasized by Shultz and Wang (2011) and Löckenhoff (2012), we need to know more about continuity and change in psychological health before, during, and following retirement. The aim of the present paper is threefold: (a) to outline the rationale of the HEalth, Ageing and Retirement Transitions in Sweden (HEARTS) study; (b) to describe the study sample, and (c) to present some initial results from the two first waves in terms of the association of retirement status and psychological health.

The rationale of the HEARTS study is rooted in several challenges linked to retirement research that have been identified in previous work, along with recent societal trends that potentially call into question results from previous studies. For example, recent changes and predictions of population dynamics, including the increased longevity and changes in perceptions and expectations of aging, (OECD, 2006; Christensen et al., 2009) call for a more comprehensive theoretical model and an updated empirical platform when it comes to aging and retirement patterns. Aging characteristics in later born cohorts differ substantially from previous generations, and the detection of life-course influences requires more detailed information about pre-aging conditions of relevance for adaptive processes related to late life outcomes. Such information is often less detailed, or even lacking, in many studies. For example, women in cohorts now entering the third age have been more engaged in the workforce which may contribute to new social patterns that need to be considered in order to understand gender differences related to the retirement transition. New cohorts also challenge previous findings due to other life-course experiences in terms of differences in education, overall lifestyle, and health-related behavior (Qi, 2016). Compared with previous generations, current cohorts of older adults face different challenges related to retirement and post-retirement life, including other experiences, expectations, and perspectives. Retirement, however, still represents a major life event. Additionally, the transition into this new period of life signals aging, both at the individual and societal level. Previously rigid regulations for a certain retirement age are gradually being replaced by more flexible systems that allow employees to withdraw pensions before or after the previous statutory retirement age of 65. Therefore, effective retirement age is increasing after the implementation of the new old-age pension system.

Previous research on the retirement transition demonstrates that continuity is the default expectation in the sense that people are “doing things to manage their transition to create continuity, contentment, or reconciliation with their new status” (see Ekerdt, 2010). At the same time, retirement requires coping and adaptation in which psychological health is challenged. Notably, the retirement transition highlights the fundamental definition of psychological aging, defined as the capacity to adapt, not only to inner biological changes but also to changes in the external environment, which can affect psychological health. There are reasons to believe that the effects of retirement transition on psychological health will vary considerably across individuals, dependent on many moderating variables. In a recent literature review (Henning et al., 2016), we show that while most retirees maintain their level of well-being over the transition, there is a substantial heterogeneity in change patterns, particularly within certain subgroups (Wang, 2007) affected by greater loss of resources that tend to compromise well-being (Pinquart and Schindler, 2007). A general finding in the review was that most previous studies failed to address change in the transition, leaving a number of critical questions unresolved.

Studies of the retirement transition face numerous methodological challenges. Labor market circumstances, type of work and job-satisfaction, personal preferences and selection issues related to health, work circumstances, socioeconomic conditions, household/family and priorities for “a good life” and life style as retiree, are only some of the internal validity factors to consider (e.g., Agahi et al., 2006; Nordenmark and Stattin, 2009). Many studies are also limited by only including men or few women. Furthermore, as proposed by Ekerdt (2010), retired life may be experienced less as an arrival but more as a personal frontier. A general observation related to psychological adaptation following retirement, is that retrospective reports tend to be more positive compared with actual pre-post ratings of health and well-being, which is a strong argument for a longitudinal design (Wells et al., 2009). This highlights the need to gather detailed pre-retirement information for an improved understanding of the retirement transition. Another challenge deals with how to conceptualize change. A temporal perspective has been called for (Shultz and Wang, 2011), which allows researchers to characterize each individual transition and examine within-person change and fluctuations on a finer scale. Previous studies have typically used data with bi-annual (e.g., Health and Retirement Study, HRS; Wang, 2007; Calvo et al., 2009), or bi-annual to less frequent (Survey of Health, Aging and Retirement Transitions in Europe, SHARE; Clark and Fawaz, 2009) measurements, resulting in large windows of time that prevent the detection of fluctuations and short-term changes around the retirement event. Studies that have included more frequent than bi-annual measurements are typically small, non-population based, and case-studies (Reitzes et al., 1996; Kim and Moen, 2002). The design, structure, and content of existing studies provide a less than optimal platform for studying the complex and rapidly changing patterns of within-person change and associations of change in different variables linked to psychological health. Thus, designs with more frequent and repeated measurements are needed.

Moreover, in most existing research on retirement, data is reported from studies (e.g., HRS or German Socio-Economic Panel) including participants who retired in the 1990s or in the 2000s, that is, between 10 and 20 years ago (the SHARE study being an exception with recent data collections 2013 and 2015). With the recent changes in retirement patterns and changing macro-conditions (Hofäcker et al., 2016), collecting updated data on retirement behavior, experiences, and the perceptions of individuals retiring today is highly relevant, both from a research perspective as well as a policy perspective.

A third challenge concerns the targeted domains of psychological health. Most previous studies have used data from: (a) large population-based studies originally designed for other research purposes than retirement; or (b) studies designed for retirement research, but with a heavy emphasis on other domains than psychological health; or a combination of (a) and (b). As a consequence, psychological health is typically measured only by single-item measures or through one concept (e.g., well-being), rather than investigated using a broader spectrum of reliable, theoretically derived multi-item variables. For example, the large and population-based German Socio-economic panel with annual measurements only includes a single-item measure of life-satisfaction (Pinquart and Schindler, 2007; Wetzel et al., 2016). As the retirement transition may have different effects on different dimensions of psychological health, a study should ideally include a variety of theoretically driven, multiple item-based, measures of psychological health. A firm consolidation and validation of findings across studies require comparisons of outcome measures in the same study (Wang et al., 2011; Muratore et al., 2014).

Finally, an important challenge for retirement researchers is how to define the concept of retirement status, and how to best measure it. While this may seem quite straightforward, it is by no means a simple task as retirement includes many different facets, can carry different meanings between individuals, and can thus be measured in different ways (Denton and Spencer, 2009). For instance, retirement is no longer viewed as a one-step permanent career exit (Wang and Shultz, 2010; Zhan and Wang, 2015). Many retirees choose to continue their work engagement in the form of bridge employments as an intermediate step toward a complete labor force withdrawal (Shultz, 2003), and today it is relatively common among older workers to retire, “un-retire,” and “re-retire” several times (Shultz and Wang, 2011; Beehr and Bennett, 2015). It is therefore important to be able to distinguish between different types of retirement statuses and transitions when studying its influence on various psychological outcomes. Thus, the very definition of retirement is far from simple and measuring an individual’s retirement status can be a complicated issue (Ekerdt and DeViney, 1990; Denton and Spencer, 2009). Retirement may look very differently, and be perceived very differently, for different individuals, and the definition can be very arbitrary (Beehr and Bowling, 2013; Cahill et al., 2013). In the end, the appropriate definition of retirement, and the answer to the question of how to best measure it, likely depends on the research question. As the HEARTS study mainly targets the retirement transition and process from a psychosocial health perspective, measuring retirement status in a way that also captures the individual’s own psychological definition and perception of retirement status is warranted.

To summarize, the requirements of studies to accurately analyze, understand and draw conclusions about the dynamics of experiences and changes in well-being across the retirement event, in combination with the lack of existing studies and data-bases (at least those reported in previous published work) meeting these requirements, calls for a new study in which the retirement transition is investigated using: (a) a design with more frequent measurements before and after the retirement event; (b) a large population-based sample for whom retirement is anticipated in the near future and likely to be of concern and on the “mental agenda”; (c) a study sample followed across multiple waves with high between-wave adherence; (d) a broad range of theoretically derived and robust multi-item-based measures related to psychological health; (d) supplement data-bases for linkage to register information on life-course information and factors such as socioeconomics, living, and workforce conditions.

The launch of the HEARTS study constitutes an attempt to adhere to such a call, potentially bridging some of the present gaps in the literature on psychological health in the retirement transition. The overall aim of HEARTS is to study psychological health in the years before and following retirement with a focus on continuity and change over the transition. The main research questions of the HEARTS study are: How does the retirement transition affect psychological health in the early phases of the third age? What contributes to continuity and changes in psychological health after retirement? Which factors moderate and mediate the effect of retirement on psychological health?

## Materials and Methods

### Design and Empirical Model

The HEARTS study is a longitudinal cohort study, following individuals annually before, during, and after the retirement event. The first data collection (baseline/first wave) was conducted in spring 2015 and the first follow-up (second wave) was conducted in spring 2016. The second follow-up (third wave) is ongoing in spring 2017. The study is scheduled to provide annual follow-up data until at least the year 2019, resulting in five measurement occasions (waves) of within-person data.

**FIGURE 1:**
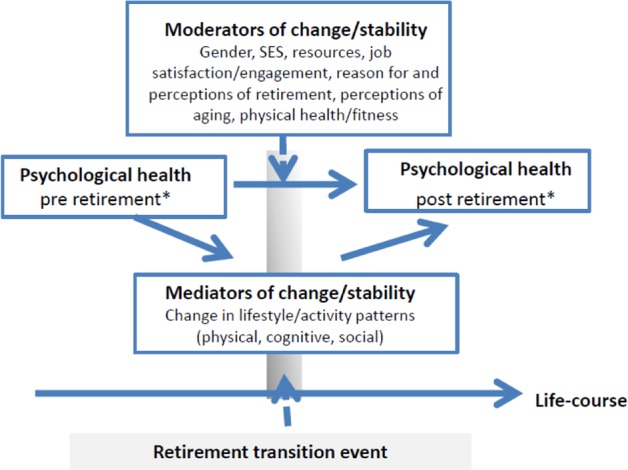
The empirical model of the HEARTS study, showing possible moderators and mediators of the effects of retirement on psychological health

A crucial assumption in the HEARTS design is that the retirement process cannot be properly characterized in terms of universal trends and statistical main effects. Instead, the retirement process is proposed to be constituted of multiple interaction effects in which different variables moderate the effect of retirement on psychological health. Following this line of reasoning, the empirical model of the HEARTS study (see **Figure 1**), highlights the role of potential moderating factors, such as gender, socioeconomic status, reason for retirement, job-satisfaction and engagement, self-perceptions and perceptions of aging, attitudes related to retirement, and physical fitness. Moreover, mediating variables are also included in the empirical model, guiding us to study the underlying mechanisms of change in psychological health across retirement. Key mediating variables in the model are overall lifestyle and activity patterns, including engagement in physical, cognitive, and social activities.

### Measurements

The HEARTS survey is divided into six parts/modules with the following themes: (i) background information (marital status, family situation, relationships, living situation); (ii) questions related to work (present or last job before retirement), retirement plans and retirement experience; (iii) health, leisure activities and health-behavior; (iv) psychological health and well-being; (v) social relations and network; (vi) personality, self-esteem and future-perspective. A more detailed description of domains, variables, and instruments used in the HEARTS study is provided in **Table 1**.

**Table 1:** Description of domains, variables, and instruments covered in the HEARTS study.

Domain	Variables	Instruments	Reference
Sociodemographic background	Gender, civil status, living context, nr of children and grand-children.	Single items	
Work life	Work demand	The Copenhagen Psychosocial Questionnaire	Pejtersen et al., 2010
	Work motivation	Multidimensional work motivation scale	Gagné et al., 2015
	Importance of performance to self-esteem	IPES	Ferris et al., 2010
Retirement	Reasons for retirement, retirement experiences and expectations	The Reasons for Retirement Questionnaire The Retirement Experiences Questionnaire	Robinson et al., 2010
Health, activities, health behaviour/ lifestyle	Self-reported health, leisure activities, psychical activity, smoking, alcohol	Single items, IPAQ, AUDIT	Saltin and Grimby, 1968; Ekelund et al., 2006
Psychological well-being	Depression	CES-D	Radloff, 1977
	Perceived stress	Perceived stress scale	Cohen and Williamson, 1988
	Life satisfaction	Satisfaction with life scale	Diener et al., 1985
	Basic psychological needs satisfaction	Basic psychological needs satisfaction	Chen et al., 2015
	Quality of life	CASP-12	von dem Knesebeck et al., 2005
	Perceived stress	Perceived stress scale	Cohen and Williamson, 1988
	Loneliness	UCLA-6	Neto, 2014
Cognitive function	Verbal abilities	Verbal scale	Based on Wechsler, 1981
	Numeracy abilities	Numeracy scale	Based on Weller et al., 2013
	Memory abilities	Memory scale	Based on Thurstone and Thurstone, 1949
	Logical thinking	Short Logical Matrices Test	Arthur and Day, 1994
	Visuo-spatial memory^∗^	Corsi Block Task	Corsi, 1972
	Executive functioning^∗^	Wisconsin Card Sorting Test	Berg, 1948
Social network	Social contacts	Lubben social network scale	Lubben, 1988
	Social support	Multidimensional scale of perceived social support	Zimet et al., 1988
Personality and attitudes	Big Five	Mini-IPIP	Donnellan et al., 2006
	Future time perspective	Future Orientation Scale	Carstensen and Lang, 1996, Unpublished
	Self-esteem	Rosenberg self-esteem scale	Rosenberg, 1965

*^∗^Only administered to a subsample in wave 1 (*n* = 382).*

In addition to the questionnaire, cognitive tests were included in the web-based survey. Different domains of cognitive performance were assessed. In both waves, memory was assessed with the original Thurstone picture memory test (Thurstone and Thurstone, 1949). Twenty pictures were subsequently presented to the participants, each one for 5 s. Then, participants were asked to identify each of the 20 previously viewed pictures when shown together with three other related, but not previously shown, pictures. In wave 1, verbal abilities were assessed using 20 questions on word knowledge, and numeracy was assessed using 12 questions. There was a 40 s time limit for each answer. An example item for assessing numeracy was: “If the chance of getting a disease is 10%, how many people would be expected to get the disease out of 1000?” (Answer alternatives: 100 people, 50 people, 10 people, 5 people, 1 person, I do not know). In wave 2, logical reasoning was measured by 12 diagrammatic puzzles, each with a missing part that the participant attempted to identify from eight options (Arthur and Day, 1994). Participants had 3 min to find all correct answers. Spatial ability was assessed in wave 2 using a test of spatial rotation (Peters and Battista, 2008). The mental rotation test requires participants to view two polygons, and judge if they are planar rotations of each other (as opposed to its mirror image).

To better capture the heterogeneity of retirement status and gradual retirement, retirement status was measured using the question “Are you retired (i.e., have started to receive old age pension)?” This question has four possible response alternatives: (a) No, (b), Yes, but still work and do not consider myself a retiree; (c) Yes, still work but consider myself a retiree; and (d) Yes, “full time retiree”. Consequently, response (a) represents individuals still working or who are unemployed whereas (b), (c) and (d) reflects retirement on a gradual scale from partial retirement/bridge retirement to full retirement, where the perception and identity of the individual linked to retirement status is most important.

Data from the HEARTS cohort will be linked to Swedish national registers containing information on mortality, disease and sociodemographic information as well as life-course information, such as cognitive status and fitness at 18 years of age for men (using the Swedish conscription data base). Our intention is to later integrate the HEARTS cohort into other ongoing Swedish longitudinal studies, creating opportunities to have more measurement points and more extended longitudinal within-person information.

### Participants

A nationally representative population-based sample of 14,990 individuals between the ages 60 and 66 was recruited in April 2015 through the register, Statens personadressregister (SPAR) in April 2015. SPAR includes all persons registered as residents in Sweden and the register is updated each day with data from the Swedish Population Register. The sample was stratified by age, but no other restrictions were made.

### Procedure

Invitations for participation in the study were sent out to 14,990 individuals. In addition to general information about the study, the invitation letter also included information on how to take part in the study through a web-based survey, administered through the Qualtrics service (web-link, individual study code and password). Non-responders received a first reminder 3 weeks later with the same information on how to answer via the web-based survey. Three weeks later a second and final reminder was sent out together with a paper-version of the survey (in addition to information that the web-link was still valid). The rationale for using a computer-based platform for annual surveys is based on the knowledge of wide internet access and computer use in Sweden, including among older adults (Findahl, 2012). However, those who prefer a paper-based questionnaire are offered this option throughout the study.

Cognitive tests were only included in the web-based survey and represent different aspects of cognitive function. We did not include reaction-time based measures, because different electronic devices and differences in internet connection speed could distort the results. The selected tests could be performed on all devices. These tests were not intended to be diagnostic, but as indicators of cognitive performance in different domains.

The HEARTS study was carried out in accordance with the recommendations of the regional ethical approval board of the University of Gothenburg with written informed consent from all subjects. All subjects gave written informed consent in accordance with the Declaration of Helsinki. Ethical approval was granted from the ethical approval board of the University of Gothenburg (Dnr: 970-14).

## Some Empirical Highlights of the Hearts Study

In addition to describing the theoretical rationale and background of the HEARTS study, in the present paper we present some empirical highlights of the two first waves of the study. These include findings concerning (a) cross-sectional differences in psychological health between individuals with different retirement status (working, partially retired or fully retired) at baseline, and (b) changes in psychological health across 1 year for different retirement status groups (working both waves, retired both waves, and retiring between waves).

### Measures Included in the Empirical Analysis

For the analyses presented in the present paper, we selected four variables representing psychological health as outcome variables: stress, depression, quality of life, and autonomy. Stress was analyzed using the Perceived Stress Scale (Cohen and Williamson, 1988). This scale includes 10 items. Participants reported how often they experienced different feelings in the last week, using a 5-point Likert-type scale. One item was: “In the last week, how often have you felt nervous and stressed?” Depression was assessed using a 10-item version of the CES-D scale (Radloff, 1977). Participants reported on a 4-point Likert-type scale (ranging from “rarely/none of the time” to “most/all of the time”) how often they experienced specific depressive symptoms in the last week. An example of an item is: “I felt depressed.” Quality of life was assessed using the CASP-12 scale (von dem Knesebeck et al., 2005). The CASP-12 includes 12 items targeting autonomy, pleasure, control, and self-realization, using a 4 point Likert-type scale. An example of an item is: “I look forward to each day.” A sum score was used instead of scoring the separate sub-dimensions. Autonomy was assessed using a sub-scale of the Basic Psychological Needs Satisfaction and Frustration Scale (Chen et al., 2015). Three items assessed autonomy on a 5-point Likert-type scale. An example of an item used is: “I feel a sense of choice and freedom in the things I undertake.”

### Analysis

In the first step, we conducted descriptive analyses to describe the HEARTS sample at baseline (wave 1) and first follow-up (wave 2) a year later. In the second step, we first used bivariate correlation analysis to test associations between the four psychological variables in wave 1 and 2 along with a reliability analysis (Cronbach’s alpha). For the main analyses, we compared the four retirement groups (described in the Measures section) in measurements of depression, stress, autonomy and quality of life, using ANOVAs, followed by Games-Howell *post hoc* tests, in SPSS. Secondly, we compared change between wave 1 and 2 in these variables, between those who were retired in both waves (still retired), those who retired between waves (retirees), and those who stayed working between waves (still working), using repeated-measure ANOVAs.

### Survey Response and Study Sample Description

In total 5,913 persons completed the first wave survey, resulting in a response rate of 39.4%. Among the participants, 4,068 (68.8%) persons responded to the web-based survey and 1,845 (31.2%) responded using the paper version (see **Table 2**). The cohort consists of slightly more women (53.0%) than men (45.4%), slightly more older individuals (aged 65–66 years), and a majority of Swedish born individuals (84.7%). The majority reported being married or having a partner (71.0%), having one or several children (88.8%), and having one or several grandchildren (65.4%). In terms of education, the cohort is better educated compared to the general population, with the largest group (31.3%) reporting having a university degree. In total, 49.6% reported completing some tertiary education level (post-secondary level).

**Table 2:** Description of the HEARTS cohort at baseline and at follow-up one year later.

Characteristics	HEARTS baseline total sample (*N* = 5913)	HEARTS baseline web-based sample (*n* = 4068)	HEARTS baseline paper sample (*n* = 1845)	HEARTS follow-up total sample (*N* = 4651)
	Frequency *n* (%)	Frequency *n* (%)	Frequency *n* (%)	Frequency *n* (%)
**Demographics**				
Gender				
Men	2683 (45.4%)	1943 (47.8%)	740 (40.1%)	2080 (44.7%)
Women	3132 (53.0%)	2055 (50.5%)	1077 (58.4%)	2507 (53.9%)
Other	1 (0.02%)	–	1 (0.1)%	1 (0.0%)
Birth year				
1955	804 (13.6%)	569 (14.0%)	235 (12.7%)	595 (12.8%)
1954	772 (13.1%)	540 (13.3%)	232 (12.6%)	602 (12.9%)
1953	814 (13.8%)	576 (14.2%)	238 (12.9%)	634 (13.6%)
1952	855 (14.5%)	588 (14.5%)	267 (14.5%)	670 (14.4%)
1951	817 (13.8%)	550 (13.5%)	267 (14.5%)	645 (13.9%)
1950	870 (14.7%)	583 (14.3%)	287 (15.6%)	711 (15.3%)
1949	912 (15.4%)	624 (15.3%)	288 (15.6%)	748 (16.1%)
**Highest level of Education**				
Did not finish primary education or shorter primary than 9 years	140 (2.4%)	72 (1.8%)	68 (3.7%)	91 (2.0%)
Finished primary education	771 (13.0%)	412 (10.1%)	359 (19.7%)	552 (11.9%)
Secondary education (Gymnasium)	1987 (33.6%)	1311 (32.2%)	676 (36.6%)	1536 (33.0%)
Community college or 2 Year College	1081 (18.3%)	799 (19.7%)	282(15.3%)	889 (19.1%)
College or University Graduate	1853 (31.3%)	1419 (34.9%)	434 (23.5%)	1533 (33.0%)
Country of birth				
Sweden	5006 (84.7%)	3598 (88.4%)	1408 (76.3%)	4035 (86.8%)
Other	647 (10.9%)	433 (10.6%)	214 (11.6%)	448 (9.6%)
Marital status				
Married/Partner	4199 (71.0%)	3011 (74.0%)	1188 (64.4%)	3380 (74.5%)
Unmarried (never been married)	488 (8.3%)	288 (7.1%)	200 (10.8%)	343 (7.6%)
Divorced/separated	836 (14.1%)	566 (13.9%)	270 (14.6%)	611 (13.5%)
Widow/Widower	225 (3.8%)	145 (3.6%)	80 (4.3%)	197 (4.3%)
Retirement status				
Not retired	3793 (64.1%)	2674 (65.7%)	1119 (60.7%)	2248 (48.3%)
Retired and working, consider myself a worker	443 (7.5%)	321 (7.9%)	122 (6.6%)	490 (10.5%)
Retired and working, consider myself a retiree	260 (4.4%)	188 (4.6%)	72 (3.9%	340 (7.3%)
Retired “full time,” not working	1263 (21.4%)	818 (20.1%)	445 (24.1%)	1522 (32.7%)

In terms of retirement status, the majority of the sample (64.1%, *n* = 3793) reported not being retired at baseline (i.e., still working or unemployed). Of the remaining individuals, 7.5% (*n* = 443) reported being retired but working and not considering themselves a retiree, 4.4% (*n* = 260) reported being retired and working, but considering themselves a retiree, and 21.4% (*n* = 1263) reported being “full time retired.”

Of those who reported not being retired, 63.7% (*n* = 2451) were working full-time, 19.2% (*n* = 725) were working part-time, 3.9% (*n* = 149) were unemployed, 2.4% (*n* = 90) were on sick leave, 6.4% (*n* = 242) received disability pensions, and 3.5% (*n* = 131) said that they were fully retired, even though they had not started to take out pensions. Information on work status was missing for 41 participants.

In the first annual follow-up in 2016 (the second wave measurement), 4,651 individuals completed the survey again, representing 78.7% of the baseline sample. Among those, 3,612 (77.7%) completed the web-survey and 1,039 (22.3%) completed the paper version. Compared to responders at follow-up, those who declined or for other reasons were non-responders at the follow-up (*n* = 1262, 21.3%) consisted of a greater proportion of men, participants in the lower age range, individuals not born in Sweden, unmarried and divorced, and participants still working. Also, individuals missing at follow-up reported lower education levels, higher scores on depression and stress, lower life-satisfaction and also scored lower in all cognitive domains (verbal, memory, and numeracy) at baseline.

In terms of change in retirement status, 12% of the baseline sample changed from working full time to being fully retired (i.e., changed from response [a] to [d]). Another 10% reported that they had changed from working full-time to receiving pension but continued working (i.e., change from [a] to [b] or [c]). Only 2% of the baseline full retirees changed back to full-time work, and another 6% had started working again to some extent while receiving pension (partial retirement).

### Retirement Status, and Psychological Health at Baseline

Correlations and reliability estimates (Cronbach’s alphas) for the four psychological health variables in wave 1 and 2 are described in **Table 3**. The reliability estimates for all measures were over.70 in both wave 1 and 2. There were moderately strong positive correlations between stress and depression and between quality of life and autonomy at both wave 1 and 2. There were also negative correlations between stress and depression with quality of life and autonomy at both waves. The longitudinal associations found between T1 and T2 were similar to those cross-sectional associations found at T1, albeit slightly weaker.

**Table 3:** Reliability estimates (Cronbach’s alpha, [α]) and correlations between the psychological health variables used in the current study at wave 1 (W1) and wave 2 (W2).

Variables	α	1.	2.	3.	4.	5.	6.	7.	8.
(1) Stress W1	0.79	–							
(2) Depression W1	0.79	0.58	–						
(3) Quality of life W1	0.85	–0.59	–0.66	–					
(4) Autonomy W1	0.74	–0.47	–0.51	0.67	–				
(5) Stress W2	0.79	0.54	0.43	–0.45	–0.39	–			
(6) Depression W2	0.80	0.45	0.64	–0.54	–0.40	0.54	–		
(7) Quality of life W2	0.86	–0.43	–0.55	0.66	0.50	–0.49	–0.56	–	
(8) Autonomy W2	0.73	–0.41	–0.44	0.57	0.63	–0.40	–0.37	0.51	–

Baseline differences in psychological health across the four retirement groups are described in **Table 4**. Individuals not yet retired and still working reported higher levels of stress compared with fully retired individuals and those still working but considering themselves retirees. Additionally, the non-retired group reported more depressive symptoms compared with all three retired groups. Individuals in the three retired groups reported higher quality of life and autonomy compared to non-retired individuals. No differences were found at baseline between the three retired groups.

**Table 4:** Differences in psychological health across the four retirement status groups at baseline.

Variables	Not retired (*n* = 3793)	Retired and working, consider myself a worker (*n* = 443)	Retired and working, consider myself a retiree (*n* = 260)	Retired “full time”, not working (*n* = 1263)	*F*-value
	M (*SD*)	M (*SD*)	M (*SD*)	M (*SD*)	
Stress^1^	22.2^a^ (5.9)	21.5^ab^(6.0)	20.8^b^ (5.5)	21.5^b^(6.1)	7.92^∗∗^
Depression^2^	4.3^a^(4.1)	3.6^b^(3.7)	3.5^b^(3.5)	3.7^b^(4.2)	11.76^∗∗^
Quality of life^3^	37.8^b^(6.2)	39.0^a^(6.0)	39.3^a^(5.3)	38.8^a^(6.3)	12.95^∗∗^
Autonomy^4^	11.2^b^(2.3)	11.9^a^(2.2)	12.1^a^(2.0)	12.1^a^(2.2)	60.98^∗∗^

*^1^Based on Perceived stress scale, scores ranging from 10 to 50; ^2^Based on the 10 item version of the CESD-D scale, scores ranging from 0 to 30; ^3^Based on CASPI-12, scores ranging from 12 to 48; ^4^Based on Basic Psychological Need Scale, scores ranging from 3 to 15; Values in the same row that do not share a common subscript (e.g., ^a,b,c^) are significantly different at *p* < 0.05 level according to the Games-Howell *post hoc* test. ^∗^*p* < 0.05, ^∗∗^*p* < 0.01.*

### Retirement Status Change and Change in Psychological Health between the First and Second Waves

**FIGURE 2:**
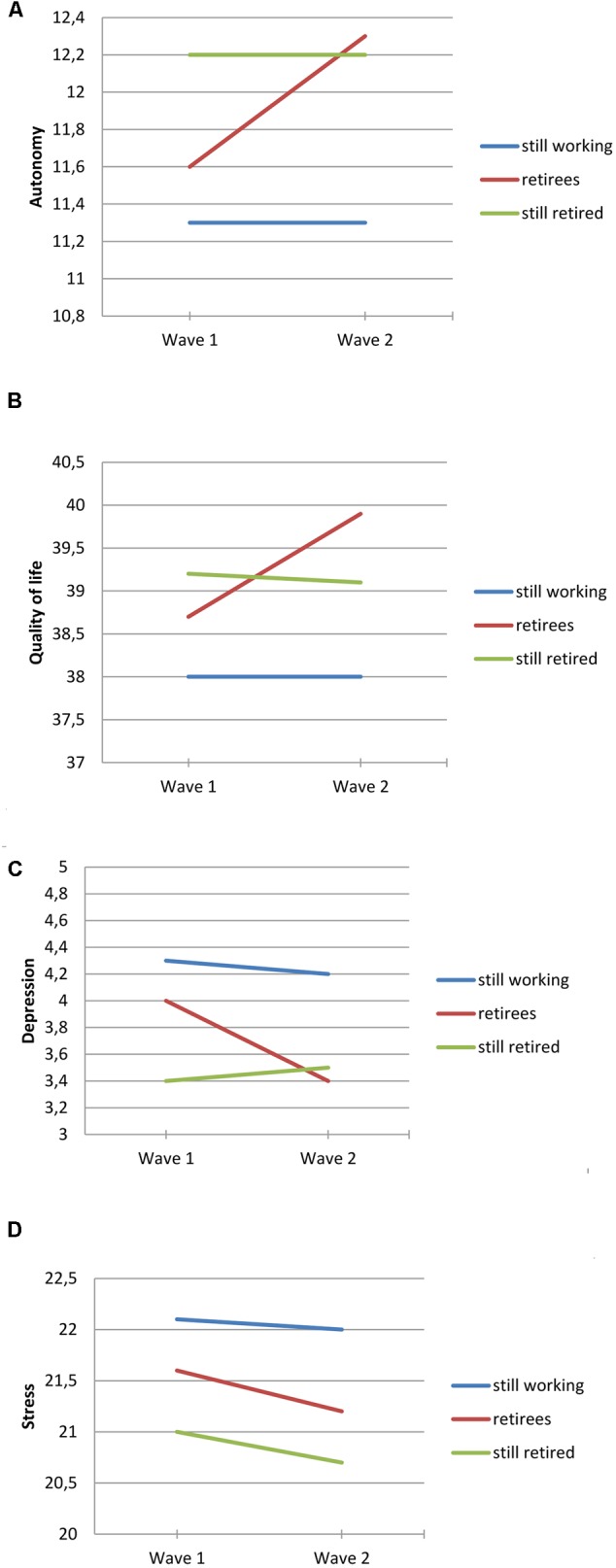
**(A–D)** Change in psychological health variables [autonomy in figure **(A)**, quality of life in figure **(B)**, depression in figure **(C)**, stress in figure **(D)**], across wave 1 and 2, comparing participants still working across waves, still being retired across waves, and retiring between waves.

Patterns of change and continuity in psychological health over the 1 year period between wave 1 and wave 2 are described in **Table 5**. Across the three retirement status groups (working at both waves, retired at both waves, or retiring between waves) there were significant Time effects for all variables. Compared to the baseline, participants at wave 2 generally reported lower stress and depression and higher quality of life and autonomy. Significant Time × Group interactions were found for all variables except for stress. The interactions are illustrated in **Figures 2A–D**. The general pattern was similar for all of these interactions (i.e., differences in retirement status change groups in change patterns). Participants working at both waves (blue lines in figures) displayed stability or a marginal increase in quality of life and autonomy, and stability or a marginal decrease in stress and depression. Participants fully retired at both waves (green lines in figures) also demonstrated a general pattern of stability in these variables. In contrast to the working group, the small change demonstrated in this group was in the opposite direction, showing small decreases in quality of life and autonomy and a small increase in depression. Participants retiring between waves (i.e., moving from retirement status categories [a] or[b] to [c] or [d]), illustrated with red lines in the figures, demonstrated a quite different overall pattern of change, with increases in quality of life and autonomy and decreases in depression between waves.

**Table 5:** Change in psychological health across baseline (first wave) and second wave for different retirement status change groups.

Variables	Full sample (*n* = 4523)	Still working (*n* = 2694)	Still retired (*n* = 1169)	Retirees (*n* = 660)	F-values Time effect/ Time × Retirement status effect
	M (SD)	M (*SD*)	M (*SD*)	M (*SD*)	
Stress T1	21.7 (5.9)	22.1 (5.9)	21.0 (5.9)	21.6 (5.6)	
Stress T2	21.5 (5.9)	22.0 (6.1)	20.7 (5.6)	21.2 (5.8)	6.93^∗^/0.94
Depression T1	4.0 (4.0)	4.3 (4.1)	3.4 (3.8)	4.0 (3.7)	
Depression T2	3.9 (4.0)	4.2 (4.1)	3.5 (3.7)	3.4 (3.8)	6.80^∗^/8.28^∗^
Quality of life T1	38.4 (6.1)	38.0 (6.1)	39.2 (6.0)	38.7 (6.1)	
Quality of life T2	38.5 (6.8)	38.0 (6.8)	39.1 (5.9)	39.9 (8.1)	9.34^∗^/11.58^∗^
Autonomy T1	11.6 (2.2)	11.3 (2.2)	12.2 (2.0)	11.6 (2.2)	52.20^∗^/35.62
Autonomy T2	11.7 (2.2)	11.3 (2.3)	12.2 (2.0)	12.3 (2.1)	

*Still working, Working both at first and second wave; Still retired, Retired in both first and second wave; Retirees, moved into retirement between wave 1 and wave 2. W1, baseline measure (first wave); W2, second wave measure (1 year post baseline). Main effects of time for full sample: Stress, *F*(1,3738) = 6.93, *p* < 0.01; Depression, *F*(1,3921) = 6.80, *p* < 0.01; Quality of life, *F*(1,3658) = 9.34, *p* < 0.01; Autonomy, *F*(1,4015) = 52.20, *p* < 0.001.*

## Discussion

The HEARTS study represents an attempt to close some of the gaps identified in previous research and to provide a better insight into the dynamics of retirement transitions, in particular linked to psychological health. A key notion highlighted in the literature on retirement (e.g., Shultz and Wang, 2011; Wang et al., 2011; Löckenhoff, 2012) is that retirement needs to be viewed as a transition process, not as a simple binary outcome. Thus, we need to consider both time-to, as well as time-from, the retirement event, which requires a longitudinal design to more accurately map and better understand the adaptive mechanisms engaged before, during, and after the retirement event. As pointed out in previous work (e.g., Shultz and Wang, 2011; Löckenhoff, 2012), little is known about the dynamics of the retirement transition when it comes to individuals’ psychological health and adaptation, and there is a gap in the understanding of what mechanisms shape individual retirement trajectories. Although there are examples of previous work in which the issue of dynamics of retirement transitions have been highlighted (e.g., Pinquart and Schindler, 2007; Wang, 2007), via large samples and sophisticated analyses, systematic knowledge on how individuals’ psychological health (using multiple indicators) are affected over the retirement transition is still lacking.

Retirement is likely accompanied by both losses and gains (Wang et al., 2011; Henning et al., 2016). Analyses therefore need to consider the negative and positive effects of the transition by accounting for the multiple factors that can modify the effects of retirement and identify the filters and lenses that act to shape post-retirement transitions. The preliminary findings from the two first waves of the HEARTS study presented in the present paper depict retirement as a generally positive experience for most individuals. Compared to working individuals, retired individuals scored higher on several of the psychological health variables (e.g., life-satisfaction, quality of life, autonomy) in addition to reporting lower stress and depression at baseline.

Our analyses on the baseline data showed that individuals who are in the middle of the retirement transition (i.e., receive old age pension but are still engaged in the workforce to some extent), reported better psychological health. These results are in line with the previously demonstrated positive effect of bridge jobs (Kim and Feldman, 2000; Zhan et al., 2009; Dingemans and Henkens, 2014; Lux and Scherger, 2017) and partial retirement (Nikolova and Graham, 2014) on health and life-satisfaction. However, the associations between bridge jobs, working in retirement, psychological health and life-satisfaction are likely more complex and influenced by a number of moderating variables, such as voluntariness and sense of control, (Calvo et al., 2009; Dingemans and Henkens, 2014; Nikolova and Graham, 2014) and satisfaction with household income (Lux and Scherger, 2017). In general, however, as noted in previous review papers (Wang and Shultz, 2010; Beehr and Bennett, 2015), very little is known about the actual outcomes of bridge employment.

Given the cross-sectional nature of the baseline data, it is also possible that the association between retirement status and psychological health mirrors the effects of the retirement position, or, selection effects into earlier retirement, or a combination of both. For example, it is possible that bridge employment engagement is partly determined by individual resources and capability (Beehr and Bennett, 2007, 2015). Having more resources, and consequently likely better psychological health, could thus increase chances to engage in bridge employment, which in turn could be beneficial for psychological health, creating a positive spiral effect (virtuous cycle effect). As such, bridge employment and working in retirement may be viewed as outcomes of individual resources and psychological health, rather than predictors of such. The more positive psychological health of the partially retired groups therefore also provides preliminary evidence for the notion of a healthy-worker effect, further highlighting the need to tease apart the directionality of the association between health and retirement behavior (Zhan et al., 2009; Burdorf, 2010; van der Heide et al., 2013).

The question of the potential positive and negative effects of retirement also taps into the basic questions of why people work, continue to work, and what functions work serves (Beehr and Bennett, 2015). Jahoda (1997), for example, describes a number of latent functions of work (aside from the manifest financial function), which are time structure, social contact, collective purpose, activity, and identity or status. All of these factors could potentially moderate the effects of retirement, or partial retirement and bridge jobs, on both levels of post-retirement health and psychological well-being, as well as changes and trajectories in these outcomes over the retirement transition. It should also be noted that retirement, that is the absence of a job, could potentially lead to some of the same functions as described above, for example a chance for increased activity and social contacts. As the HEARTS study includes a broad spectrum of measurements linked to these proposed functions, data from future waves will afford the possibility to examine these assumptions more rigorously, using change-analyses on both between- and within-person levels, as well as a combination of variable and person-centered analyses.

The way of defining and measuring retirement status in the HEARTS study, using two categories to capture partial retirement, targeting primarily the individual perception of partial retirement (identifying oneself as a worker or retiree when working after retirement), will hopefully better account for the variance in retirement behavior, compared with the traditional dichotomous definitions typically used in previous work (i.e., only distinguishing between workers and retirees). We believe that the differentiation between various types of retirement status and transitions may be an important step forward in understanding its influence on psychological outcomes. For instance, although it is generally believed that a more gradual transition is beneficial for the retirement adjustment process (Cahill et al., 2013), we still lack in our understanding of the underlying mechanisms of how and why retirees may or may not benefit from different types of retirement transition (Zhan and Wang, 2015). The emphasis on individual perception (to what degree do I see myself as a retiree?) when measuring retirement status in HEARTS is also naturally related to the heavy focus on psychosocial outcomes, forming a logic link between independent and dependent variables in the study. By including a more subjective component, it is possible to investigate to what extent changes in psychological health can be understood as an effect of “subjective” or “objective” retirement. As this specific way of targeting retirement has not, to our knowledge, been used in previous studies, it is not possible at this stage to draw any firm conclusions in terms of how the measurement and definition of retirement itself has affected the results presented in the present paper.

The longitudinal findings confirm the notion that retirement has a positive impact on psychological health. Individuals retiring between waves demonstrated a positive change in psychological health, such as increase in life-satisfaction, quality of life and autonomy and a decrease in depression. Participants still working or still retired showed a more stable trend. Although only two measurement points across 2 years provide a limited platform for drawing conclusions about patterns of long-term post-retirement trajectories, these results may mirror the “honeymoon” effect documented in previous studies (e.g., Wetzel et al., 2016). Interestingly, this effect does not seem to be limited to single variables (Wang, 2007; Dingemans and Henkens, 2014) or even one-item based single variables of psychological well-being (Pinquart and Schindler, 2007; Wetzel et al., 2016) as has been documented in previous work, but is evidently valid for a number of the conceptually different variables of psychological health included in HEARTS. Our demonstration of such broad effects on various concepts of psychological well-being contributes with novel insights into the very nature of retirement and how broadly the retirement transition affects individuals The stability patterns of already retired individuals may reflect a “disenchantment” phase in Atchley’s model of stages of retirement, following the honeymoon (see Atchley, 1976; Reitzes and Mutran, 2004), further pointing to the notion that retirement patterns and processes may follow a complex non-linear change trend for many individuals.

The overall aim of the HEARTS study is to investigate psychological health in the years before and following retirement with a focus on continuity and change over the transition. The major research questions that will guide future analyses are: How does the retirement transition affect psychological health in the early phases of the third age? Is lifestyle affected and do changes in adaptive processes, such as lifestyle, “travel together” with changes in psychological health from the pre- to the post-retirement period? What contributes to continuity and changes in psychological health after retirement? Which factors moderate and mediate the effect of retirement on psychological health? How do factors such as gender, education, personality, personal control, social embeddedness, and physical health interact with adaptive and coping strategies related to psychological health after retirement? What are the effects of more distal life course factors, for example health, cognition and fitness earlier in life? Can we identify individuals, prior to their retirement, who are at risk for compromised psychological health post-retirement? What characterizes those individuals who exhibit a positive transition (i.e., maintained or increased levels of psychological health) into retirement compared to those who show more negative patterns?

Future waves of the HEARTS study will enable thorough testing of these research questions and the assumptions in the theoretical model. Such analyses include longitudinal mediation models (Selig and Preacher, 2009), addressing mechanisms underlying the effect of retirement on psychological health. For example, in the model, lifestyle (physical, social and intellectual activity) constitutes a key variable of interest, presumably mediating part of the effect of retirement on psychological health. Additionally, a number of moderating variables are described in the model, such as gender, socioeconomic status, work status prior to retirement, reason for retirement, and health, to name a few. Therefore, moderation analyses and conditional process analyses will be used, in which questions about moderation and moderated mediation can be tested (Hayes, 2013; Hayes and Preacher, 2013). Furthermore, in order to address heterogeneity in the population, to identify and understand subpopulations, and to highlight the effect of within-person interactions between multiple variables (e.g., personality, work motivation and lifestyle) on retirement adaptations, we will use person-centered analyses, such as latent profile analyses and growth mixture analyses (for an overview, see Wang and Hanges, 2011).

The design and data collected in the HEARTS study comprise both strengths and limitations. Using a web-based survey design can be considered a strength of the study and has produced a number of benefits. For example, data is quickly available for analyses and the data collection is less expensive compared to other alternatives. Additionally, the HEARTS cohort consists of a narrow age-span (age interval 60–66) sample that will be followed frequently (annually), which increases the ability to detect within-person changes and complex associations of within-person change before and after the retirement transition. The response rate at the first annual follow-up was almost 80%, which coupled with the annual follow-up design, should also be considered a strength of the study. Moreover, compared to existing large longitudinal studies from which data are drawn for studies on retirement transition, HEARTS comprises a wide range of theoretically driven, robust and validated measurements of psychological and psychosocial health, lifestyle and activities. Finally, the planned link between HEARTS-data and other registers and ongoing studies can be seen as a unique asset given the possibility to link later life data to unique life-course information^1^. Our planned annual longitudinal data collection will provide opportunities to examine within-person change and cross-lagged effects more carefully and grant a more stable platform for an even more detailed picture of the losses and gains in the transition, as well as the selection mechanisms that operate in the retirement context. In terms of weaknesses, as in most cohort studies, despite efforts to recruit a nationally representative sample, the HEARTS cohort is more highly educated compared to the general population, which will affect how results can be generalized. Furthermore, we found systematic differences between responders and non-responders (i.e., individuals that dropped out before wave 1 and 2) at wave 2. This pattern is not uncommon for longitudinal studies on aging (van Beijsterveldt et al., 2002; Rabbitt et al., 2004; Chatfield et al., 2005), and might lead to distorted effects. This problem will need to be addressed in future publications, using appropriate methods (Hogan et al., 2004; Ghisletta and Spini, 2004). Another weakness is that only web-based survey participants were able to perform the cognitive measures. Additionally, data collected thus far mainly consists of self-reports, aside from the cognitive tests. However, future analyses will allow us to supplement and merge this information with register data, and thereby further increase the value of the HEARTS study.

## Author Contributions

ML and BJ proposed the study. ML drafted the first draft of the paper, was main responsible for the paper and conducted the analyses. ML, BJ, AIB, PB, SB, IH, LH, GH, MK, SK, and VT were responsible for the study setup and data collection and gave input to the paper throughout the process.

## Conflict of Interest Statement

The authors declare that the research was conducted in the absence of any commercial or financial relationships that could be construed as a potential conflict of interest.

## Footnotes

1 The HEARTS data will be available for sharing and collaboration starting in the spring of 2018. Information about the study and cohort, along with a file containing the complete list of existing variables, can be found on the HEARTShomepage: www.psy.gu.se/hearts. An abstract of intent can be also downloaded on the homepage. Before receiving access to the data, interested collaborators need to complete this abstract, describing the purpose of their planned analyses and which HEARTS variables will be used, and send it to hearts@psy.gu.se. This can be done even before the data is available. More specific questions concerning the HEARTS study and the available data can be sent to magnus.lindwall@gu.se.
